# Fusion of KATZ measure and space projection to fast probe potential lncRNA-disease associations in bipartite graphs

**DOI:** 10.1371/journal.pone.0260329

**Published:** 2021-11-22

**Authors:** Yi Zhang, Min Chen, Li Huang, Xiaolan Xie, Xin Li, Hong Jin, Xiaohua Wang, Hanyan Wei

**Affiliations:** 1 School of Information Science and Engineering, Guilin University of Technology, Guilin, China; 2 Guangxi Key Laboratory of Embedded Technology and Intelligent System, Guilin University of Technology, Guilin, China; 3 School of Computer Science and Technology, Hunan Institute of Technology, Hengyang, China; 4 Academy of Arts and Design, Tsinghua University, Beijing, China; 5 The Future Laboratory, Tsinghua University, Beijing, China; 6 Pharmacy School, Guilin Medical University, Guilin, China; University of Science and Technology Liaoning, CHINA

## Abstract

It is well known that numerous long noncoding RNAs (lncRNAs) closely relate to the physiological and pathological processes of human diseases and can serves as potential biomarkers. Therefore, lncRNA-disease associations that are identified by computational methods as the targeted candidates reduce the cost of biological experiments focusing on deep study furtherly. However, inaccurate construction of similarity networks and inadequate numbers of observed known lncRNA–disease associations, such inherent problems make many mature computational methods that have been developed for many years still exit some limitations. It motivates us to explore a new computational method that was fused with KATZ measure and space projection to fast probing potential lncRNA-disease associations (namely KATZSP). KATZSP is comprised of following key steps: combining all the global information with which to change Boolean network of known lncRNA–disease associations into the weighted networks; changing the similarities calculation into counting the number of walks that connect lncRNA nodes and disease nodes in bipartite graphs; obtaining the space projection scores to refine the primary prediction scores. The process to fuse KATZ measure and space projection was simplified and uncomplicated with needing only one attenuation factor. The leave-one-out cross validation (LOOCV) experimental results showed that, compared with other state-of-the-art methods (NCPLDA, LDAI-ISPS and IIRWR), KATZSP had a higher predictive accuracy shown with area-under-the-curve (AUC) value on the three datasets built, while KATZSP well worked on inferring potential associations related to new lncRNAs (or isolated diseases). The results from real cases study (such as pancreas cancer, lung cancer and colorectal cancer) further confirmed that KATZSP is capable of superior predictive ability to be applied as a guide for traditional biological experiments.

## Introduction

Long non-coding RNAs (lncRNAs) whose length are longer than 200 nucleotides (nt) have crucial roles in gene expression control during developmental and differentiational processes [1]. Therefore, there is no surprise that mutation and dysregulation of lncRNAs could contribute to the development of various human complex diseases [2], such as HOTAIR in breast cancer [3] and MALAT1 in early-stage non-small cell lung cancer [4]. LncRNAs can also drive many important cancer phenotypes through their interactions with other cellular macromolecules including DNA, protein, and RNA [5–8]. There is urgent need to discern potential functional roles of lncRNAs to further study the pathology, diagnosis, therapy, prognosis, prevention of human complex diseases, and detect disease biomarkers at lncRNA level [9, 10]. With strong data support from lncRNA related databases (such as LncRNAdb [11], LncRNADisease [12], NRED [13], and NONCODE [14]) and similarity calculation based on miRNA information [15–20], the computational prediction models that were built to infer lncRNA–disease associations could supply more accurate targeted candidates [21]: 1) saving cost and time for biological experiments; 2) making bio-experiments focus on deeper study of targets; 3) speeding up understanding the pathogenesis of complex diseases.

The computational models used for inferring lncRNA–disease associations have been divided into three main categories: 1) Machine learning-based inferring models use naive Bayesian classifier model [22, 23], support vector machine (SVM) [24, 25], matrix completion [26, 27], matrix factorization [28–30] to infer potential lncRNA–disease associations. However, the models categorized to this category are not able to achieve high predictive accuracy. 2) Network-based inferring models, based on the biological premise that lncRNAs with similar functions tend to be associated with similar diseases [31, 32], use random walk [33–35], KATZ measure [36, 37], hyper geometric distribution [15], label propagation algorithm [38], propagating information streams [39], lncRNA-miRNA interaction [15, 30] to identify potential lncRNA–disease associations. Nevertheless, the models categorized to this category rely heavily on the information integrated from diverse biological data sources, and it is difficult to integrate heterogeneous data from multiple sources deeply. 3) Convolutional neural network (CNN) based inferring models [40–43], are at the early research stage, with consuming relatively high time complexity and relying on the quality of multiple sources biological data as well. Therefore, those above models still have different limitations, such as, needing negative samples, not being able to infer associations related to isolated diseases and new lncRNAs directly, not high accuracy with singular methodology. Addressing these limitations, we explored a novel prediction method based on the fusion of KATZ Measure and Space Projection to infer potential lncRNA-disease associations in bipartite graphs, namely KATZSP.

KATZ measure such a graph-based computational method could be used to transform the problem of calculating similarities between nodes to link prediction in bipartite graph. In the context of lncRNA-disease association prediction, the heterogeneous networks are represented by matrices (also called bipartite graph). Therefore, calculating similarities between the nodes of lncRNAs and diseases is further transformed into the problem of counting the number of walks that connect the interactive lncRNA-disease pairs in bipartite graph. Furthermore, the number of walks as the lengths decided the potential association probability of this lncRNA-disease pair [36, 44]. Space projection method [45, 46] could improve the lncRNA-disease association predictive ability easily with few regulation parameters, even though the known lncRNA-disease associations exist inherent data sparsity. After simplified and uncomplicated fusion process, KATZ measure and space projection method were fused to form an integrated computational model KATZSP with needing only one attenuation factor, while dropping above limitations.

## Experimental evaluation and discussion

### Evaluation metrics

Leave One Out Cross Validation (LOOCV) experiments were implemented for evaluating the predictive performance of KATZSP. We divided the dataset of known associations into two parts: the testing subset and the training subset. In the testing subset, each known association was used as a test data in turn, and the remaining known associations formed the training subset. Under the framework of LOOCV, we compared the prediction results on some specific threshold to obtain the following four metrics: true positive (TP), false positive (FP), false negative (FN), true negative (TN). Furthermore, according to some specified thresholds, we calculated the true positive rate ($TPR=\frac{TP}{TP+FN}$) against false positive rate ($FPR=\frac{FP}{TN+FP}$) with which to plot out the receiver operating characteristic curve (ROC). The area under the ROC curve (AUC) was finally calculated to numerically evaluate the overall predictive performance of KATZSP.

### Impact with parameter selection

**Fig 1:**
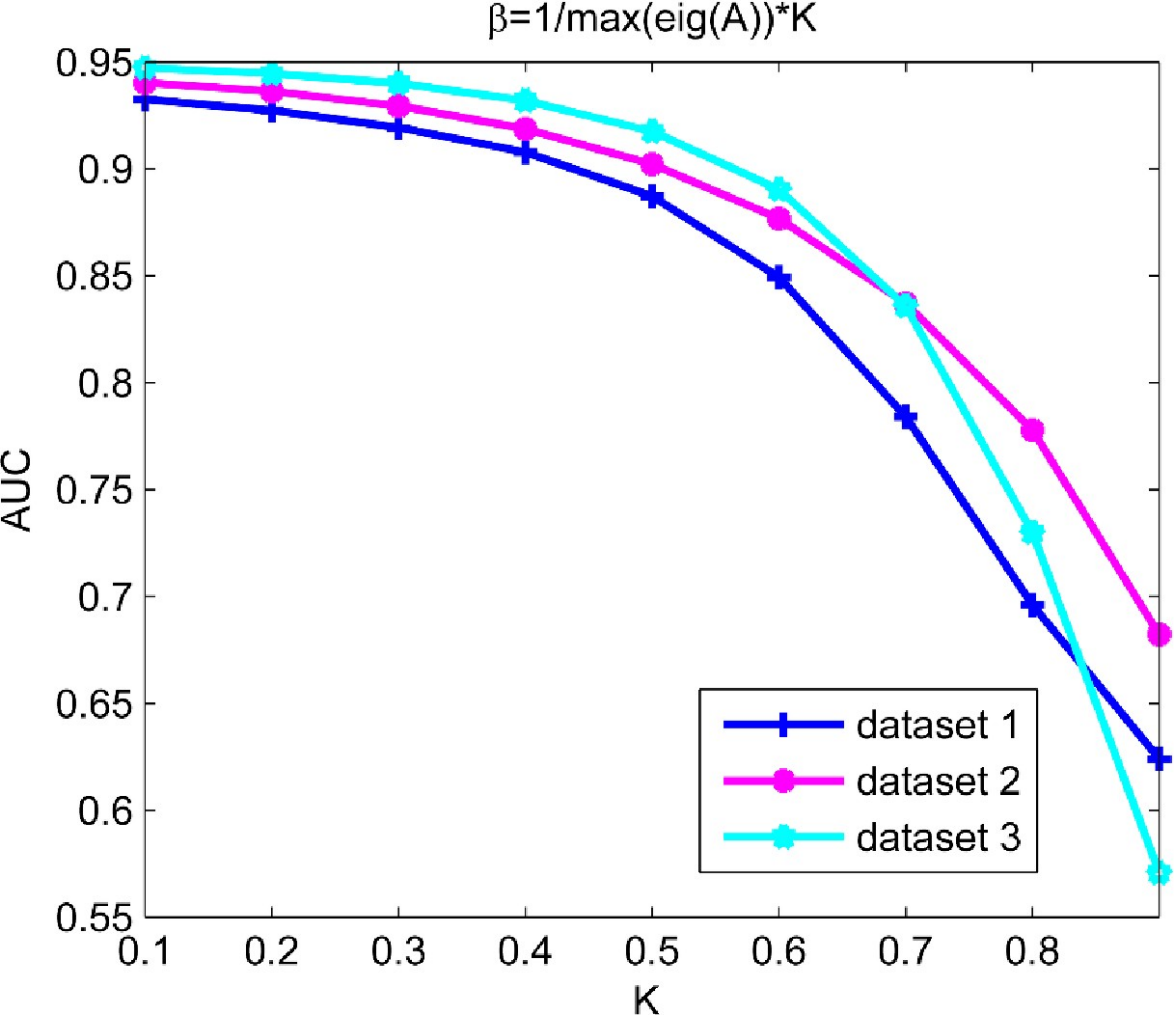
Impact with parameter variation on model prediction accuracy.

Coefficient *β* plays as an attenuation factor of weight to control the contribution of lengths coming from walks on calculating the similarities between any two interactive nodes. According to the convergence properties of sequences required by KATZ method, the value of *β* should be less than the reciprocal of the max-eigenvalue of the adjacency matrix **A**. In order to obtain the optimal value of *β*, we set *β* = 1/max(eig(**A**))*K where max(eig(**A**)) denotes the max-eigenvalue of adjacency matrix **A**. Then the value of *K* was increased from 0.1 to 0.9 with step size of 0.1. With changing the value of *K*, LOOCV was implemented on all the three datasets built (dataset 1, dataset 2 and dataset 3). The results in Fig 1 showed that AUC could achieve the maximum value on all the three datasets when *K* = 0.1.

### Compare predictive abilities under different solutions

**Fig 2:**
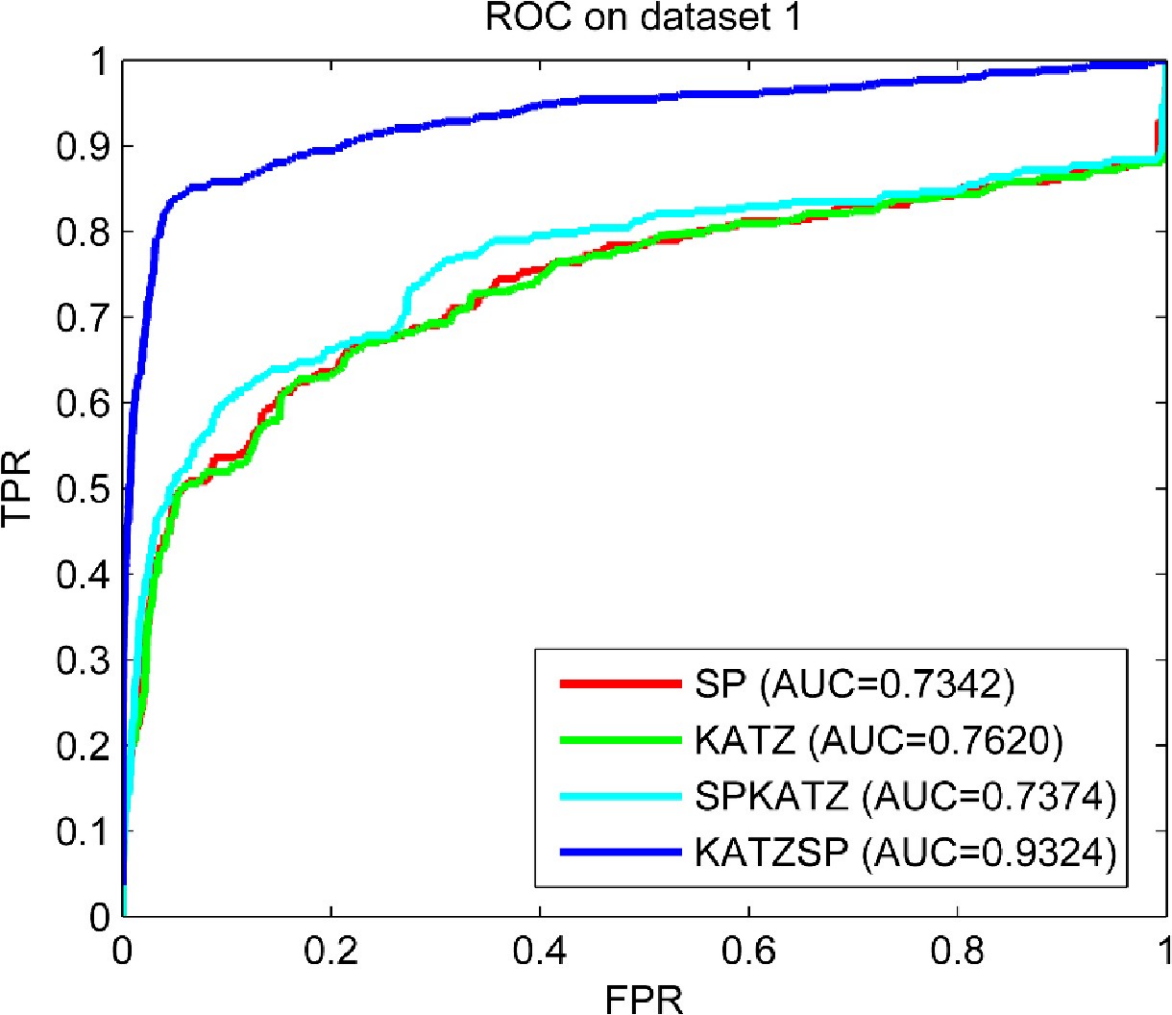
Predictive abilities with different technical solutions on dataset 1.

**Fig 3:**
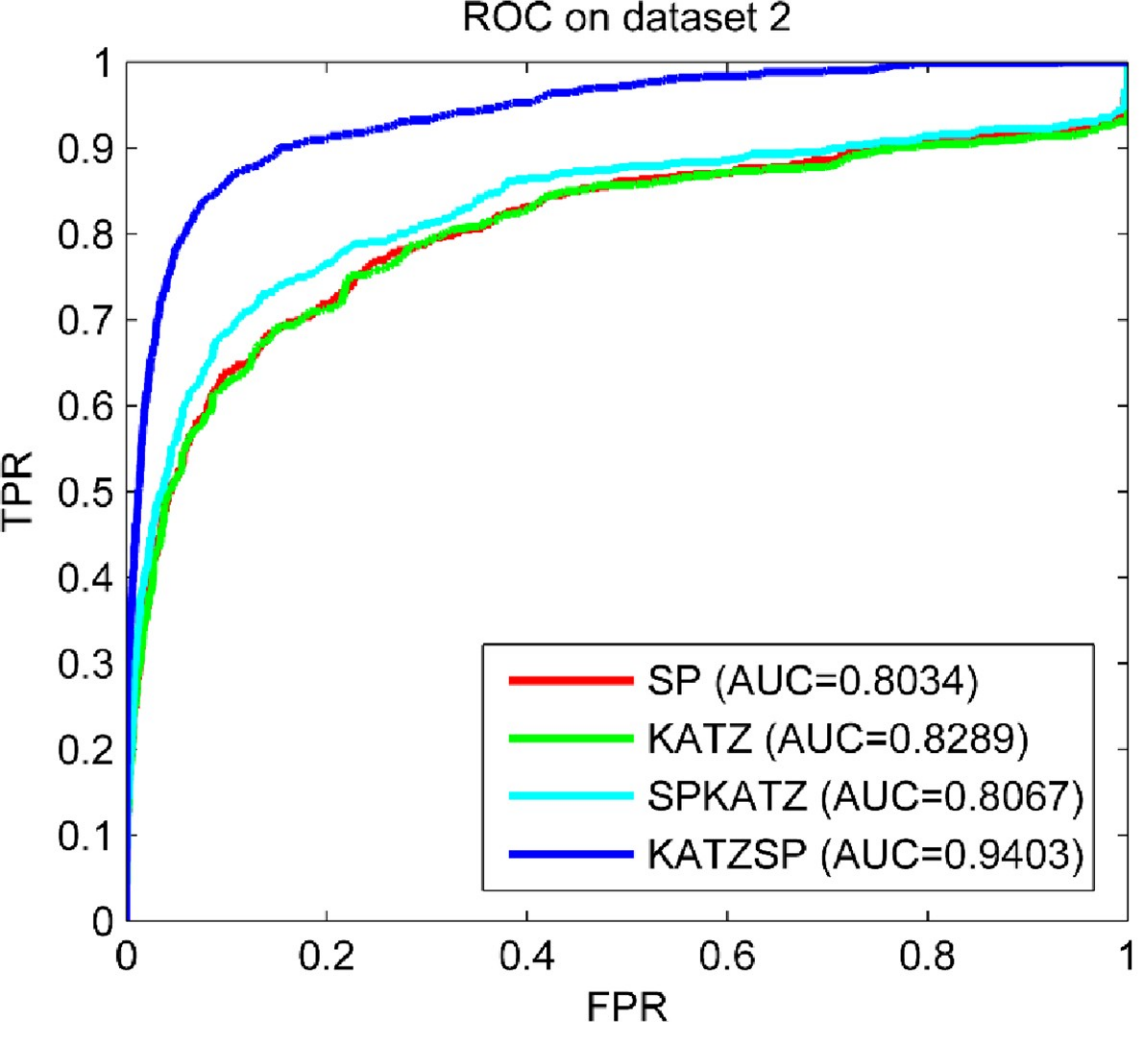
Predictive abilities with different technical solutions on dataset 2.

**Fig 4:**
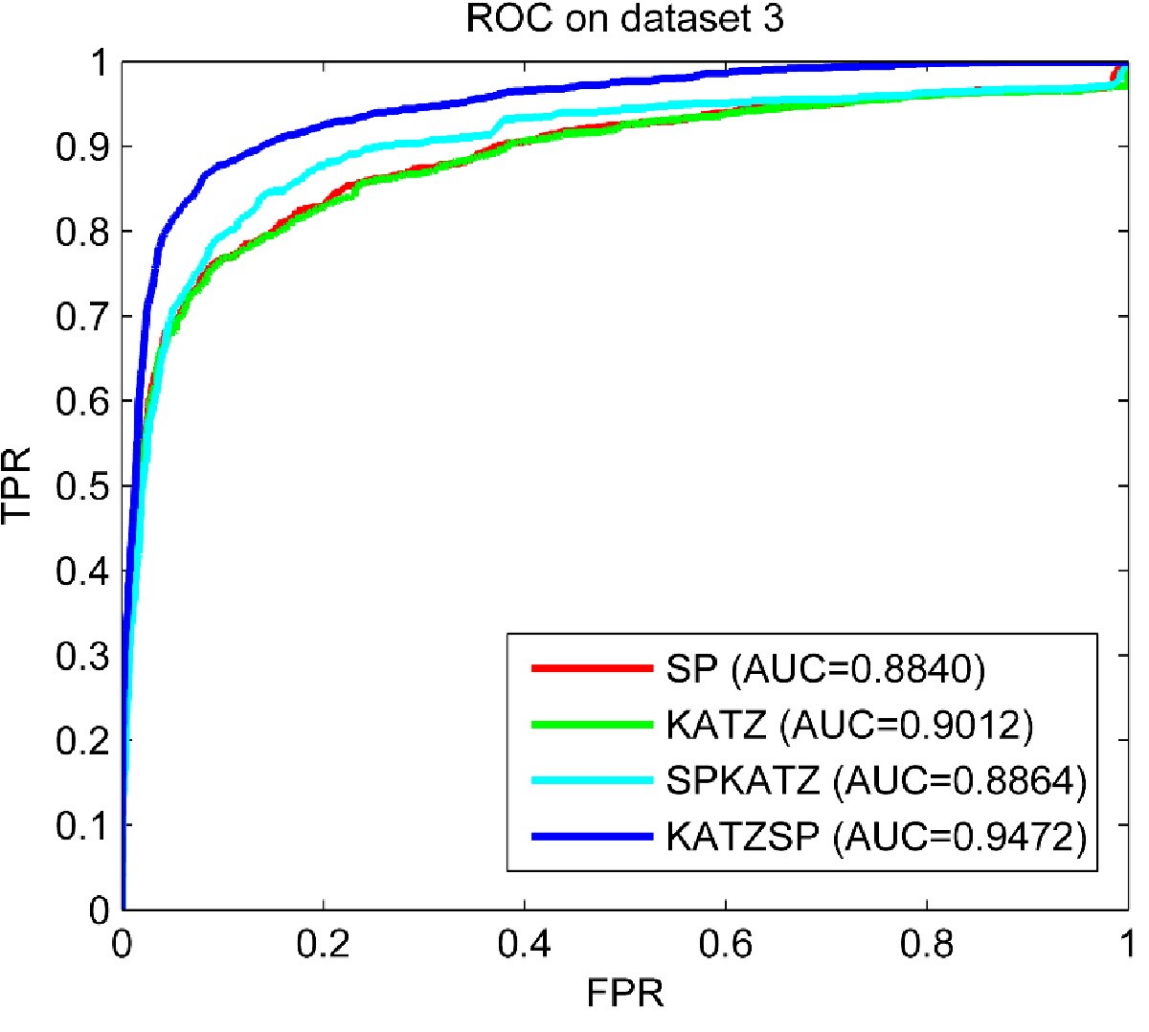
Predictive abilities with different technical solutions on dataset 3.

To demonstrate how our technical solution selected performed better than others, LOOCV experiments were implemented under following four technical solutions: only using space projection (SP), only using KATZ (KATZ), using space project first and then KATZ (SPKATZ), using KATZ first and then space projection (KATZSP). The results compared on three datasets (dataset 1, dataset 2 and dataset 3) were shown in Figs 2–4, respectively.

From the comparison results shown in Figs 2–4, we easily found the solution used in our model (KATZSP) achieved AUC values of 0.9324, 0.9403 and 0.9472 on dataset 1, dataset 2 and dataset 3, respectively. Among above four solutions, our KATZSP which performed the best predictive ability on all three datasets with distinct advantage than other three solutions.

### Compare performance with other models

**Fig 5:**
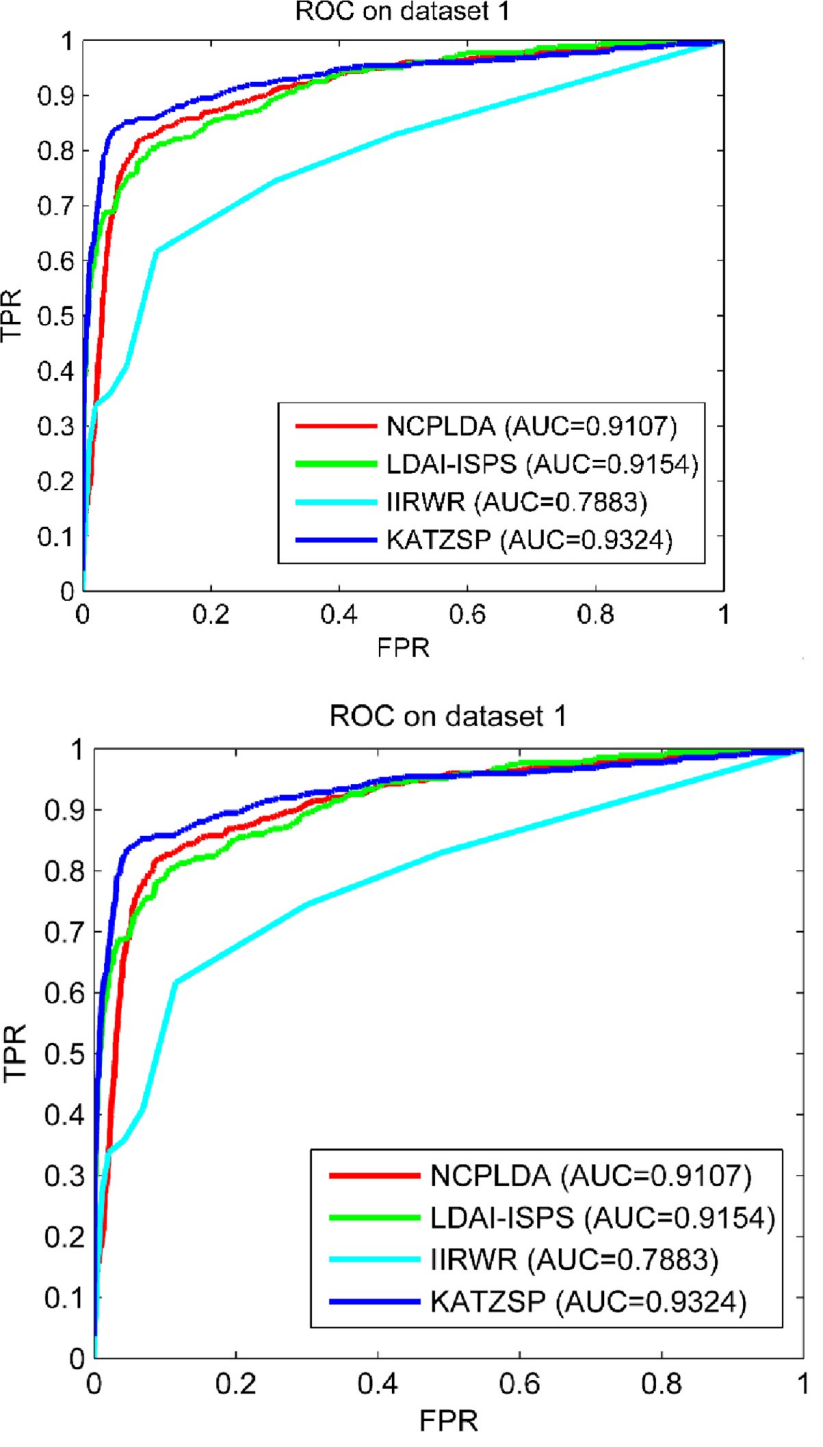
Predictive abilities of KATZSP and other models on dataset 1.

**Fig 6:**
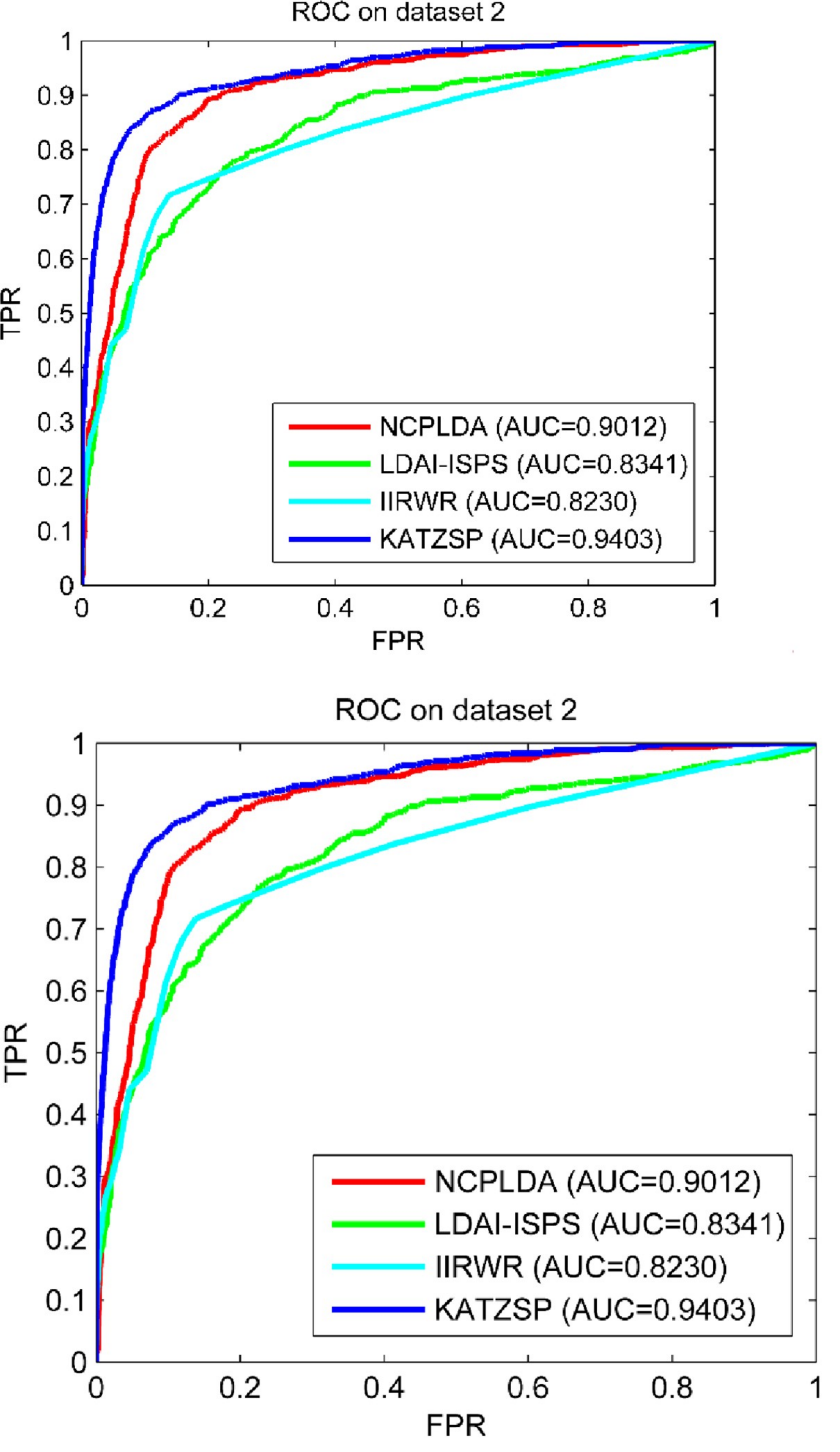
Predictive abilities of KATZSP and other models on dataset 2.

**Fig 7:**
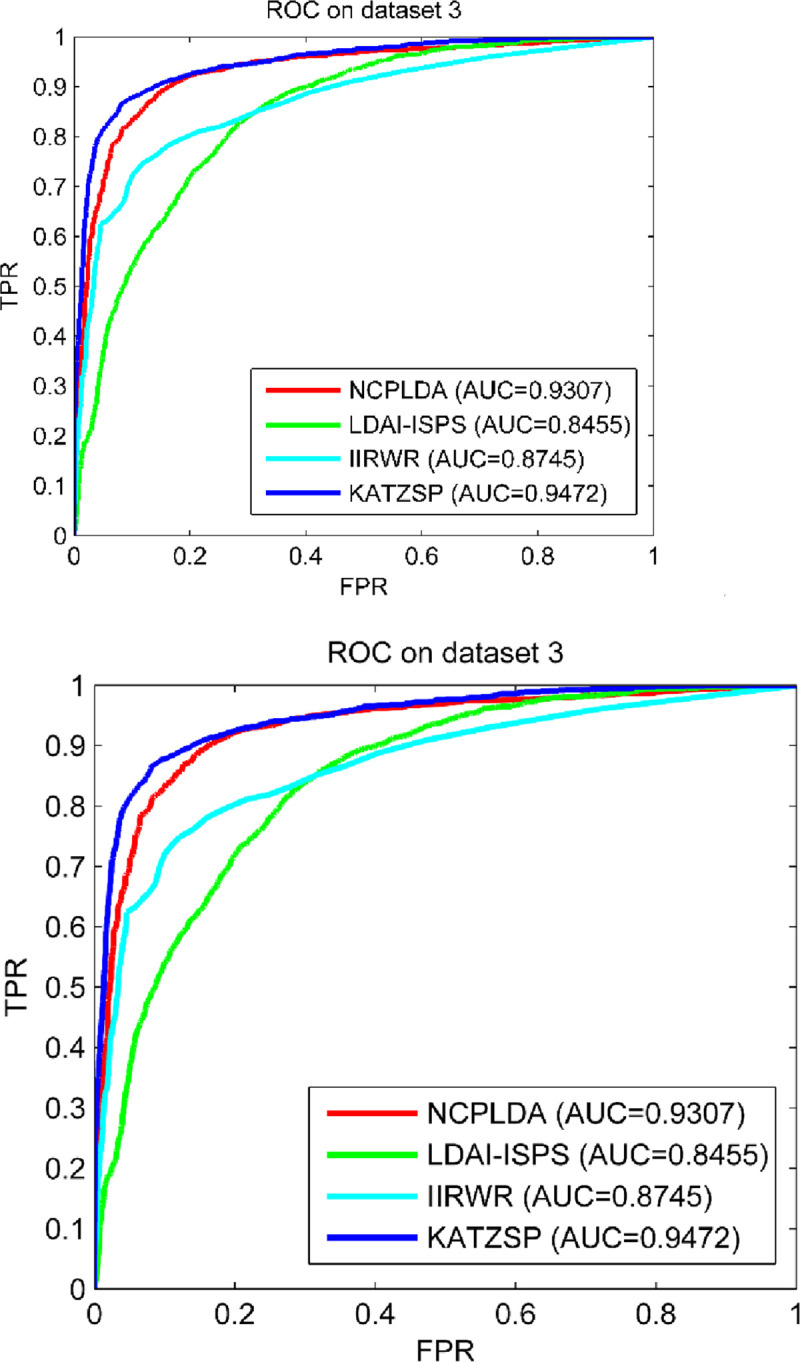
Predictive abilities of KATZSP and other models on dataset 3.

To further demonstrate the reliable predictive ability of our model, we chose some the-state-of-art computational models in similar type (NCPLDA [47], LDAI-ISPS [48] and IIRWR [49]) to compare with our model in the framework of LOOCV. To make comparison fairly, we configured the same experimental environment and condition for all models on dataset 1, dataset 2 and dataset 3. From the comparison results shown in Figs 5–7, our KATZSP achieved the highest AUC values on all three datasets with detail analysis shown in Table 1.

**Table 1:** AUCs of KATZSP and other models on all three datasets.

Model AUC value	NCPLDA	LDAI-ISPS	IIRWR	KATZSP
AUC on dataset 1	0.9107 (2.3%)	0.9154 (1.9%)	0.7883 (18.3%)	0.9324
AUC on dataset 2	0.9012 (4.3%)	0.8341 (11.8%)	0.8230 (14.3%)	0.9403
AUC on dataset 3	0.9307 (1.7%)	0.8455 (12%)	0.8745 (8.3%)	0.9472

From data of “AUC on dataset 1” in Table 1, our KATZSP was demonstrated with higher AUC values which were 2.3%, 1.9% and 18.3% higher than that of NCPLDA, LDAI-ISPS and IIRWR, respectively. Similarly, the comparison results on dataset 2 demonstrated the AUC values of our KATZSP were 4.3%, 11.8% and 14.3% higher than that of NCPLDA, LDAI-ISPS and IIRWR, respectively. In the last row of Table 1, the 1.7%, 12% and 8.3% higher AUC values of our KATZSP were compared with that of NCPLDA, LDAI-ISPS and IIRWR, respectively. Therefore, our KATZSP was demonstrated with more reliable predictive ability over other previous models on all the three datasets under the evaluation framework of LOOCV.

### Verify predictive ability for new lncRNAs and isolated diseases

**Fig 8:**
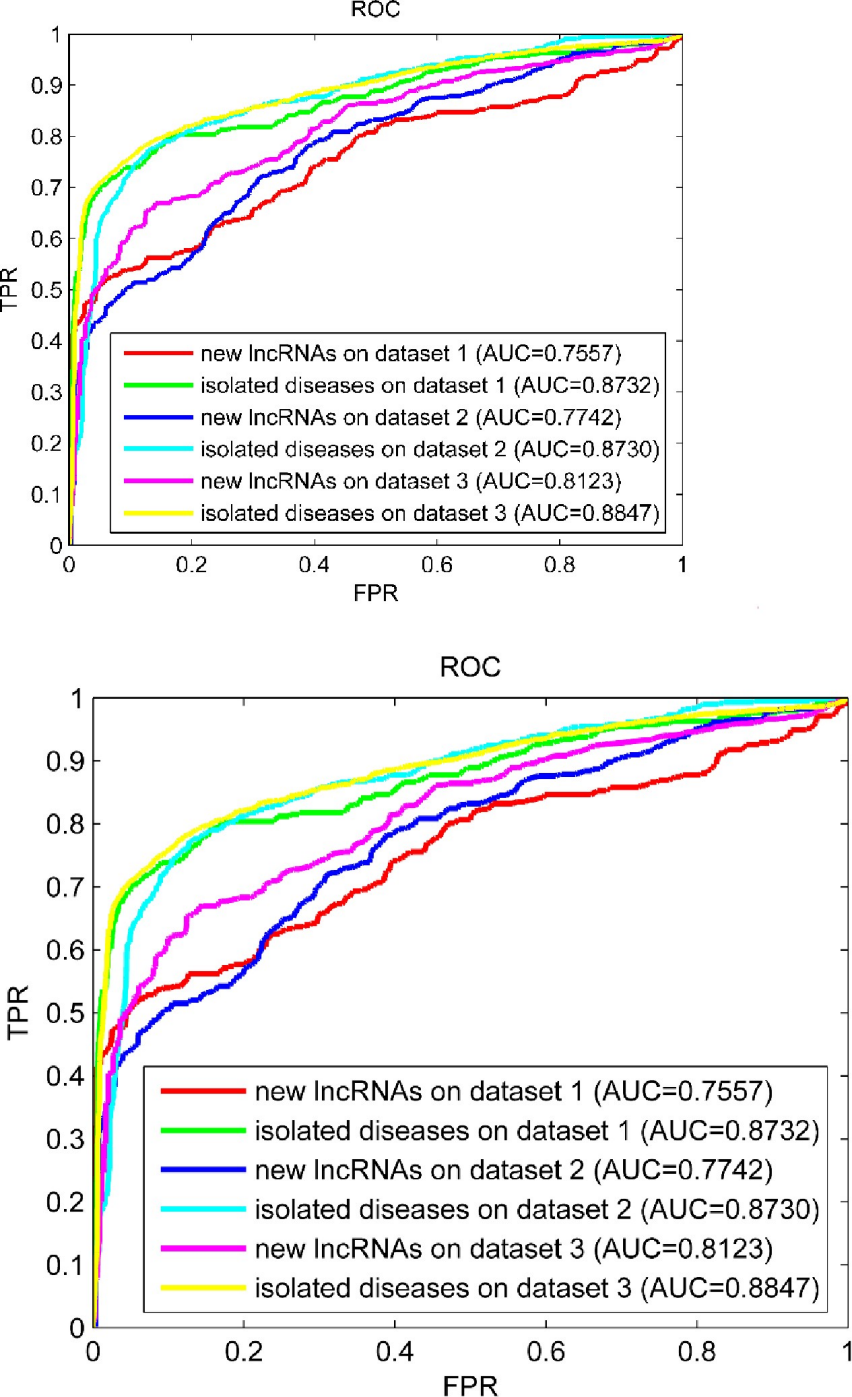
Predictive ability of KATZSP for new lncRNAs and isolated diseases.

To implement the verification in this section, we simulated each lncRNA in the known lncRNA-disease associations dataset to be a new lncRNA by removing all known associations relating to it. Similarly, we simulated each disease in the known lncRNA-disease associations dataset to be an isolated disease by removing all known associations relating to it. Each new lncRNA (or isolated disease) simulated was specified to be the test sample for model evaluation and the rest lncRNAs (or diseases) in the known lncRNA-disease associations dataset worked as the training samples for model learning. Until the associations between each new lncRNA and diseases or the associations between lncRNAs and each isolated disease were inferred by our KATZSP, the inferred results on dataset 1, dataset 2 and dataset 3 were shown in Fig 8.

With the AUC values in Fig 8, it demonstrated that our KATZSP could be effectively applied to infer associations related to new lncRNAs and associations related to isolated diseases.

## Cases study

### Case study for three specific diseases

To further demonstrate the predictive performance of our KATZSP on real cases study, we selected three specific diseases (pancreas cancer, lung cancer and colorectal cancer) as the cases to examine. With using the training samples composed of the known associations in dataset 2 and the testing samples composed of the unknown associations, our KATZSP focused on inferring the potential lncRNAs relating to above three cases. The lncRNAs with the top five highest prediction scores of each case were listed in Table 2. If the same associations predicted by KATZSP were also found in some literatures or the newest databases, such as LncRNADisease 2.0 (http://www.rnanut.net/lncrnadisease) and Lnc2Cancer 3.0 (http://www.biobigdata.net/lnc2cancer), it could further validate with the supporting evidences that our KATZSP was capable of the reliable predictive ability and practicability.

**Table 2:** Top 5 specific diseases-related candidate lncRNAs.

Case	LncRNA	Evidences	Rank
Pancreas cancer	H19	LncRNADisease	1
Pancreas cancer	MEG3	LncRNADisease	2
Pancreas cancer	CDKN2B-AS1	LncRNADisease	3
Pancreas cancer	GAS5	LncRNADisease	4
Pancreas cancer	UCA1	LncRNADisease	5
Lung cancer	PVT1	LncRNADisease	1
Lung cancer	GAS5	LncRNADisease	2
Lung cancer	CDKN2B-AS1	LncRNADisease	3
Lung cancer	UCA1	LncRNADisease	4
Lung cancer	NPTN-IT1	Lnc2Cancer	5
Colorectal cancer	PVT1	LncRNADisease	1
Colorectal cancer	CDKN2B-AS1	Lnc2Cancer	2
Colorectal cancer	LSINCT5	Lnc2Cancer	3
Colorectal cancer	GAS5	Lnc2Cancer	4
Colorectal cancer	UCA1	LncRNADisease	5

The data in column “Evidences” of Table 2 showed that all the potential lncRNAs inferred relating to the three specific diseases have been found the evidence in LncRNADisease 2.0 or Lnc2Cancer 3.0. It validated the reliability of the inferred results coming from our KATZSP.

### Case study for isolated diseases

In recent years, many new diseases without any known association r lncRNAs have been gradually discovered, namely isolated diseases. It is important to verify if our KATZSP could be applied to infer the potential lncRNAs associated to such kind of isolated diseases. Above three cases (pancreas cancer, lung cancer and colon cancer) were simulated as the isolated diseases by removing all known associations relating to them in dataset 2. Our KATZSP only used other information to infer the potential lncRNAs associated with these three isolated diseases simulated. The top five lncRNAs with highest prediction scores of each disease were listed in Table 3 where only two prediction results (TC0101441 and KRASP1) couldn’t be found supporting evidence from any databases or published literatures.

**Table 3:** Top 5 specific isolated diseases-related candidate lncRNAs.

Disease	lncRNA name	Evidences	Rank
pancreas cancer	HOTAIR	LncRNADisease	1
pancreas cancer	MALAT1	LncRNADisease	2
pancreas cancer	H19	LncRNADisease	3
pancreas cancer	MEG3	LncRNADisease	4
pancreas cancer	TC0101441	No evidence	5
lung cancer	HOTAIR	LncRNADisease	1
lung cancer	MALAT1	LncRNADisease	2
lung cancer	H19	LncRNADisease	3
lung cancer	MEG3	LncRNADisease	4
lung cancer	PVT1	LncRNADisease	5
colon cancer	HOTAIR	LncRNADisease	1
colon cancer	MALAT1	LncRNADisease	2
colon cancer	H19	LncRNADisease	3
colon cancer	EPB41L4A-AS1	Literature [50]	4
colon cancer	KRASP1	No evidence	5

In Tables 2 and 3, all predicted results except two were confirmed with extra evidences, which validated our KATZSP could be effectively applied in real life with supplying calculated candidates to guide biological experiments.

## Materials and methods

### Obtain data source

#### Known lncRNA-disease associations

From a publicly accessible address at http://www.cuilab.cn/lncrnadisease, three versions of the databases which consist of associations between lncRNAs and human diseases were obtained for our work. With processing of the database in version 2013, we built a new dataset (namely dataset 1) with 352 known lncRNA–disease associations involved in 156 lncRNAs and 190 diseases. With processing of the database in version 2016, a new-built dataset (namely dataset 2) consists of 621 known lncRNA–disease associations involved in 285 lncRNAs and 226 diseases. With processing of the database in version 2017, a similar new-built dataset (namely dataset 3) consists of 1695 known lncRNA–disease associations involved in 828 lncRNAs and 314 diseases. The observed lncRNA–disease associations with lncRNA nodes and disease nodes form the bipartite graph denoted by the Boolean matrix **LD** = (*ld*_*ij*_)_*nl*×*nd*_, whose element *ld*_*ij*_ is 1 when lncRNA *l*_*i*_ relates to disease *d*_*j*_. Otherwise, the value of element *ld*_*ij*_ is 0. The number of lncRNAs and the number of diseases in matrix **LD** are denoted by *nl* and *nd*, respectively.

#### Disease–disease semantic similarity

Referring to the description by Wang et al. [51], in DAG (Directed Acyclic Graph), the contribution of a disease *d*_*t*_ to the semantics of disease *d*_*i*_ has following definition with denotation of $D_{d_{i}}(d_{t})$:

$$D_{d_{i}}(d_{t})=\{\begin{array}{c} 1,\;\mathrm{if}\;d_{t}=d_{i}\\ \max\{\Delta *D_{d_{i}}(d_{t^{\prime }})|d_{t^{\prime }}\in \mathrm{children}\;\mathrm{of}\;d_{t}\},\;\mathrm{if}\;d_{t}\neq d_{i} \end{array}$$ (1)

where Δ was set to be the most suitable value of 0.5.

Based on both the addresses of diseases in DAG graphs and the semantic relations with ancestor diseases, the element *dd*_*ij*_ in matrix **DD** = (*dd*_*ij*_)_*nd*×*nd*_ denotes the semantic similarity between diseases *d*_*i*_ and *d*_*j*_ with definition as follows:

$$dd_{ij}=\frac{\sum_{d_{t}\in T_{d_{i}}\cap T_{d_{j}}}\left(D_{d_{i}}(d_{t})+D_{d_{j}}(d_{t})\right)}{\sum_{d_{t}\in T_{d_{i}}}D_{d_{i}}(d_{t})+\sum_{d_{t}\in T_{d_{j}}}D_{d_{j}}(d_{t})}$$ (2)

where $T_{d_{i}}$ is the set of all ancestor nodes relating to disease *d*_*i*_, including node *d*_*i*_ itself in DAG.

#### LncRNA–lncRNA functional similarity

How to accurately measure the functional similarity between two lncRNAs was detailly descripted in many literatures [47–49, 52]. A group of diseases which have associations with lncRNA *l*_*i*_ were denoted by $D^{\left(l_{i}\right)}=\{d_{i_{1}},d_{i_{2}},\cdots ,d_{i_{k}}\}$, and the similarity between any disease *d*_*t*_ in $D^{\left(l_{i}\right)}$ and the whole set $D^{\left(l_{i}\right)}$ has following definition:

$$S(d_{t},D^{\left(l_{i}\right)})=\underset{1\leq x\leq k}{\max}dd_{ti_{x}}$$ (3)

Similarly, set $D^{\left(l_{j}\right)}=\{d_{j_{1}},d_{j_{2}},\cdots ,d_{j_{k^{\prime }}}\}$ denotes a group of diseases associate with lncRNA *l*_*j*_. The similarity between any disease *d*_*t*_ in $D^{\left(l_{j}\right)}$ and the whole set $D^{\left(l_{j}\right)}$ has following definition:

$$S(d_{t},D^{\left(l_{j}\right)})=\underset{1\leq x\leq k^{\prime }}{\max}dd_{tj_{x}}$$ (4)

Functional similarities between the lncRNAs were denoted by **LL** = (*ll*_*ij*_)_*nl*×*nl*_ whose element *ll*_*ij*_ represents the functional similarity between *l*_*i*_ and *l*_*j*_ with calculation as follows:

$$ll_{ij}=\frac{\sum_{1\leq x\leq k}S(d_{i_{x}},D^{\left(l_{j}\right)})+\sum_{1\leq y\leq k^{\prime }}S(d_{j_{y}},D^{\left(l_{i}\right)})}{k+k^{\prime }}$$ (5)

#### Central similarity of the Gaussian interaction profile

Compared to the number of unknown lncRNA–disease associations, the number of known lncRNA–disease associations is very small, which leads the bipartite graph represented by Boolean matrix of known lncRNA–disease associations to have sparsity. In order to reduce the influence from sparsity on prediction precision, the central similarities of Gaussian interaction profile were calculated in accordance with the description in Laarhoven’s work [53]. Therefore, the central similarities of Gaussian interaction profile between the diseases were denoted by $DD^{\left(g\right)}={\left(dd_{ij}^{g}\right)}_{nd\times nd}$ whose element $dd_{ij}^{g}$ represents the central similarity of Gaussian interaction profile between disease *d*_*i*_ and *d*_*j*_ with following definition:

$$dd_{ij}^{g}=\exp(-\gamma_{d}{\left\|LD(:,i)-LD(:,j)\right\|}^{2})$$ (6)

where the *i*th column of matrix **LD** was denoted by **LD**(:,*i*) which represents all the known associations relating to disease *d*_*i*_; The Gaussian kernel bandwidth here was denoted by *γ*_*d*_ with following definition in accordance to the previous study [54]:

$$\gamma_{d}=\frac{1}{\frac{1}{nd}\sum_{i=1}^{nd}{\left\|LD(:,i)\right\|}^{2}}$$ (7)

Similarly, the central similarities of Gaussian interaction profile between the lncRNAs were denoted by $LL^{\left(g\right)}={\left(ll_{ij}^{g}\right)}_{nl\times nl}$ whose element $ll_{ij}^{g}$ represents the central similarity of Gaussian interaction profile between lncRNA *l*_*i*_ and *l*_*j*_ with definition as follows:

$$ll_{ij}^{g}=\exp(-\gamma_{l}{\left\|LD(i,:)-LD(j,:)\right\|}^{2})$$ (8)

where the *i*th row of matrix **LD** was denoted by **LD**(*i*,:) which represents all the known associations relating to lncRNA *l*_*i*_; The Gaussian kernel bandwidth here was denoted by *γ*_*l*_ with following definition:

$$\gamma_{l}=\frac{1}{\frac{1}{nl}\sum_{i=1}^{nl}{\left\|LD(i,:)\right\|}^{2}}$$ (9)

#### Integrated similarity of lncRNAs and diseases

The final similarity matrix of diseases denoted by $DD^{\left(f\right)}={\left(dd_{ij}^{f}\right)}_{nd\times nd}$ comes from an integration of **DD** and **DD**^(*g*)^, and the final similarity matrix of lncRNAs denoted by $LL^{\left(f\right)}={\left(ll_{ij}^{f}\right)}_{nl\times nl}$ comes from an similar integration of **LL** and **LL**^(*g*)^. When the original semantic similarity between disease *d*_*i*_ and *d*_*j*_ was 0, the value of element $dd_{ij}^{f}$ in matrix **DD**^(*f*)^ was set as the central similarity of the Gaussian interaction profile, otherwise it was set as the original semantic similarity between disease *d*_*i*_ and *d*_*j*_. The value of element $ll_{ij}^{f}$ in matrix **LL**^(*f*)^ has a similar setting process as above. For clarity, the formalized acquirement for element values was defined as follows:

$$dd_{ij}^{f}=\{\begin{array}{l} dd_{ij},\;\mathrm{if}\;dd_{ij}\neq 0\\ dd_{ij}^{g},\;\mathrm{otherwise} \end{array}$$ (10)

$$ll_{ij}^{f}=\{\begin{array}{l} ll_{ij},\;\mathrm{if}\;ll_{ij}\neq 0\\ ll_{ij}^{g},\;\mathrm{otherwise} \end{array}$$ (11)

### Obtain primary prediction scores

#### Construct adjacency matrix

Based on KATZ measurement, the number of walks that connect lncRNA nodes and disease nodes in the original bipartite graph were calculated to measure the similarities between these nodes as the potential association probabilities. The different lengths of walks between lncRNA nodes and disease nodes contributed differently to the similarities between these two kinds of nodes. The shorter length of walks contributed more to the similarities than the longer one. To make full use of the heterogeneous network constructed above, matrix **DD**^(*f*)^, **LL**^(*f*)^ and **LD** were integrated into a new heterogeneous network **A**_(*nl*+*nd*)×(*nl*+*nd*)_ as the adjacency matrix with definition as follows:

$$A=[\begin{array}{cc} LL^{\left(f\right)} & LD\\ LD^{T} & DD^{\left(f\right)} \end{array}]$$ (12)

#### Calculate primary prediction score on KAZT measurement

By applying KATZ measurement, potential association probabilities between node *l*_*i*_ and node *d*_*j*_ could be calculated as follows with denotation of $S^{KATZ}(l_{i},d_{j})$:

$$S^{KATZ}(l_{i},d_{j})=\sum_{w=1}^{m}\beta^{w}{\left(A^{w}\right)}_{l_{i},d_{j}}$$ (13)

where *β* is a non-negative coefficient to control the contribution of lengths coming from walks on the similarities between any two nodes, such as *l*_*i*_ and *d*_*j*_, *β*^*w*^ raised to the power of *w*, ${\left(A^{w}\right)}_{l_{i},d_{j}}$ denotes the number of paths whose length of walks equals *w* between corresponding nodes pair, such as *l*_*i*_ and *d*_*j*_, *m* denotes the maximum value of the length of walks.

Because bigger value of the length of walks contributes less to the similarities between two nodes, the above formula for similarity calculation could be approximately described in matrix when the value of *m* tends to be infinity (*m*→∞):

$$S^{KATZ}=\sum_{w=1}^{\infty }\beta^{w}A^{w}=\sum_{w\geq 1}\beta^{w}A^{w}={\left(I-\beta A\right)}^{-1}-I$$ (14)

where the value of coefficient *β* was set in range of (0,min{1,1/‖**A**‖_2_}), matrix *S*^*KATZ*^ has the same size as adjacency matrix **A**.

Submatrix *S*^*KATZ*^[1:*nl*,*nl*+1:*nl*+*nd*] denotes the elements that located at the rows 1 to *nl* and the columns *nl*+1 to *nl*+*nd* in matrix *S*^*KATZ*^, which has the same location as matrix **LD** in adjacency matrix **A**. In order to express in a consistent way, submatrix *S*^*KATZ*^[1:*nl*,*nl*+1:*nl*+*nd*] was denoted by matrix $LD_{nl\times nd}^{\left(p\right)}=(ld_{ij}^{p}{)}_{nl\times nd}$ to represent the primary prediction results in the first stage.

### Refine primary prediction scores

In order to improve the prediction performance of the proposed model, matrix space projection was used to refine the primary prediction scores obtained in the first stage ($LD_{nl\times nd}^{\left(p\right)}$).

#### Project on lncRNA space

Project the final similarity matrix of lncRNAs (**LL**^(*f*)^) on the matrix of primary prediction scores (**LD**^(*p*)^) to obtain the projection scores on the lncRNA space, which were denoted by $LD_{nl\times nd}^{\left(pl\right)}={\left(ld_{ij}^{pl}\right)}_{nl\times nd}$ with detailed definition as follows:

$$ld_{ij}^{pl}=\frac{LL^{\left(f\right)}(i,:)\times LD^{\left(p\right)}(:,j)}{\left\|LD^{\left(p\right)}(:,j)\right\|}$$ (15)

where $ld_{ij}^{pl}$ denotes the predicted score of the association between lncRNA *l*_*i*_ and disease *d*_*j*_ with lncRNA space projection, ‖**LD**^(*p*)^(:,*j*)‖ is the 2-norm of vector **LD**^(*p*)^(:,*j*).

#### Project on disease space

Similarly, project the final similarity matrix of diseases (**DD**^(*f*)^) on the matrix of primary prediction scores (**LD**^(*p*)^) to obtain the projection scores on the disease space, which were denoted by $LD_{nd\times nl}^{\left(pd\right)}={\left(ld_{ij}^{pd}\right)}_{nd\times nl}$ with detailed definition as follows:

$$ld_{ij}^{pd}=\frac{DD^{\left(f\right)}(j,:)\times {\left(LD^{\left(p\right)}(i,:)\right)}^{T}}{\left\|LD^{\left(p\right)}(i,:)\right\|}$$ (16)

where (**LD**^(*p*)^(*i*,:))^*T*^ denotes the transpose of vector **LD**^(*p*)^(*i*,:), and ‖**LD**^(*p*)^(*i*,:)‖ is the 2-norm of vector **LD**^(*p*)^(*i*,:).

#### Integrate space projection scores

In order to fully capture the information of disease similarity, lncRNA similarity, and known lncRNA–disease associations, we integrated the projection scores on lncRNA space ($LD_{nl\times nd}^{\left(pl\right)}$) and the projection scores on disease space ($LD_{nd\times nl}^{\left(pd\right)}$) to obtain the final prediction scores ($LD_{nl\times nd}^{\left(f\right)}$) with detailed definition as follows:

$$LD^{\left(f\right)}=\frac{LD^{\left(pl\right)}+{\left(LD^{\left(pd\right)}\right)}^{T}}{2}$$ (17)

### Represent workflow model

**Fig 9:**
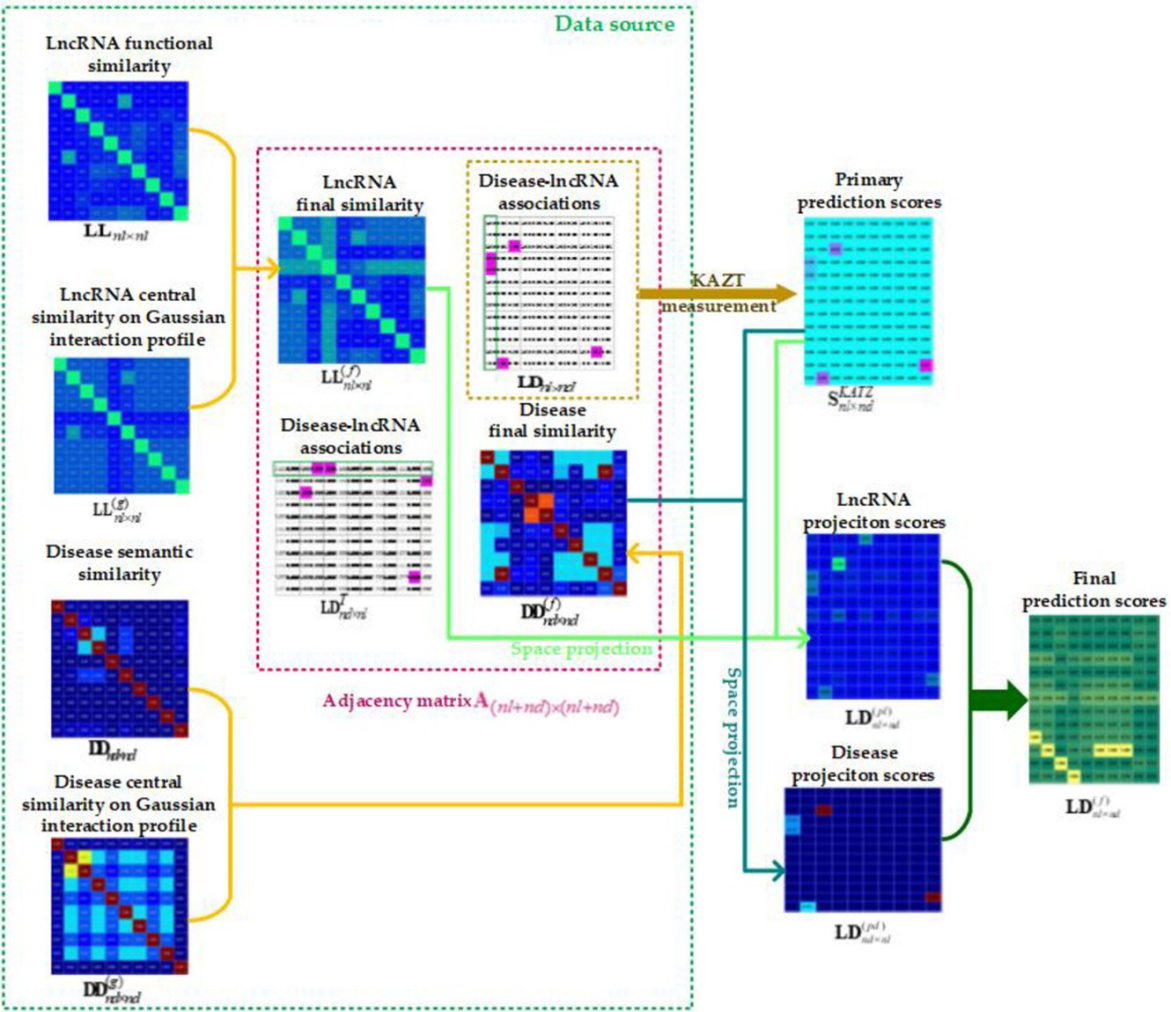
Workflow model of KATZSP.

With the related data preparation, the inferring process with each key step of KATZSP for lncRNA-disease associations was graphically reprensented in Fig 9.

## Conclusions

In recent years, even though many computational models for inferring lncRNA–disease associations have emerged, those computational methods still have some limitations that motivated us to propose a new model (KATZSP) to infer lncRNA–disease associations. The main contribution of KATZSP is composed of: only needing one attenuation factor *β* to control the contribution of walk lengths between any two nodes in bipartite graphs; making up the sparsity with simply integrating KATZ measurement and space projection; no needing negative samples; being able to be applied to isolated diseases and new lncRNAs directly. Compared with some state-of-the-art methods in similar type (NCPLDA, LDAI-ISPS and IIRWR), our model KATZSP achieved higher prediction accuracy on all three datasets (dataset 1, dataset 2 and dataset 3). The results from case study further confirmed the stronger predictive performance of KATZSP to be applied for real cases. Our KATZSP still has following limitations that need to be improved in future: further reducing the biases that the predicted results prefer the data with more known associations; the prediction accuracy needing to be enhanced further with fusion of more heterogeneous data.

## Supporting information

S1 File We have released our code publicly at the address of https://github.com/zywait/KATZSP. In the public repository released includes our minimal underlying datasets (data352.mat, data621.mat, data1695.mat).
(ZIP)

## Abbreviations

LOOCV: leave-one-out cross validation
ROC: receiver operating characteristic
AUC: area under the ROC curve
FPR: false positive rate
TPR: true positive rate
